# Risk of Brain Tumors in Children and Susceptibility to Organophosphorus Insecticides: The Potential Role of Paraoxonase (PON1)

**DOI:** 10.1289/ehp.7680

**Published:** 2005-03-18

**Authors:** Susan Searles Nielsen, Beth A. Mueller, Anneclaire J. De Roos, Hannah-Malia A. Viernes, Federico M. Farin, Harvey Checkoway

**Affiliations:** ^1^Public Health Sciences Division, Fred Hutchinson Cancer Research Center, Seattle, Washington, USA; ^2^Department of Epidemiology and; ^3^Department of Environmental and Occupational Health Sciences, School of Public Health and Community Medicine, University of Washington, Seattle, Washington, USA

**Keywords:** brain tumor, children, chlorpyrifos, diazinon, dried blood spots, Guthrie cards, paraoxonase, pesticides, PON1, xenobiotic metabolism

## Abstract

Prior research suggests that childhood brain tumors (CBTs) may be associated with exposure to pesticides. Organophosphorus insecticides (OPs) target the developing nervous system, and until recently, the most common residential insecticides were chlorpyrifos and diazinon, two OPs metabolized in the body through the cytochrome P450/paraoxonase 1 (PON1) pathway. To investigate whether two common *PON1* polymorphisms, C-108T and Q192R, are associated with CBT occurrence, we conducted a population-based study of 66 cases and 236 controls using DNA from neonatal screening archive specimens in Washington State, linked to interview data. The risk of CBT was nonsignificantly increased in relation to the inefficient *PON1* promoter allele [per *PON1*_-108T_ allele, relative to *PON1*_-108CC_: odds ratio (OR) = 1.4; 95% confidence interval (CI), 1.0–2.2; *p*-value for trend = 0.07]. Notably, this association was strongest and statistically significant among children whose mothers reported chemical treatment of the home for pests during pregnancy or childhood (per *PON1*_-108T_ allele: among exposed, OR = 2.6; 95% CI, 1.2–5.5; among unexposed, OR = 0.9; 95% CI, 0.5–1.6) and for primitive neuroectodermal tumors (per *PON1*_-108T_ allele: OR = 2.4; 95% CI, 1.1–5.4). The Q192R polymorphism, which alters the structure of PON1 and influences enzyme activity in a substrate-dependent manner, was not associated with CBT risk, nor was the *PON1*_C-108T/Q192R_ haplotype. These results are consistent with an inverse association between PON1 levels and CBT occurrence, perhaps because of PON1’s ability to detoxify OPs common in children’s environments. Larger studies that measure plasma PON1 levels and incorporate more accurate estimates of pesticide exposure will be required to confirm these observations.

Some epidemiologic studies have observed increased risk of childhood brain tumors (CBTs) in relation to home pesticide use, farm residence, or parental occupation in agriculture (Bunin et al. 1994; Cordier et al. 2001; Davis et al. 1993; Efird et al. 2003; Holly et al. 1998; Kristensen et al. 1996; Pogoda and Preston-Martin 1997). Organophosphorus insecticides (OPs) target the nervous system, and it is possible that CBT occurrence is associated with prenatal or childhood exposure to OPs or a reduced ability to metabolize them. One important OP-detoxifying enzyme is paraoxonase 1 (PON1). Present in the liver and blood, PON1 hydrolyzes the acetylcholinesterase-inhibiting oxons (activated intermediates) of some OPs, including chlorpyrifos and diazinon (Costa et al. 2002), which are important in agriculture and in recent decades were the most common insecticides used in homes and yards (Donaldson et al. 2002). Although some environmental exposures influence PON1 activity (Costa et al. 2005), they appear to have much less effect than genetic variation (Jarvik et al. 2002); thus, PON1 levels are relatively stable after they reach adult levels at 6–15 months of age (Cole et al. 2003).

The *PON1* gene contains several common single nucleotide polymorphisms, some of which directly affect OP metabolism. In the promoter region, adjacent to a binding site for the transcription factor Sp1 (Deakin et al. 2003), the C-108T polymorphism influences expression of the gene, with the *PON1*_-108T_ allele conferring reduced PON1 levels. It is the most influential known polymorphism in the promoter region, contributing 22–25% of variation in PON1 expression in white adults (Brophy et al. 2001; Leviev and James 2000). Relative to *PON1*_-108CC_ homozygotes, *PON1*_-108TT_ homozygotes have, on average, 33–45% lower enzyme activity as adults (Brophy et al. 2001; Leviev and James 2000) and 63% lower as neonates (Chen et al. 2003). PON1’s OP detoxification activity is also influenced by enzyme structure, and a coding region polymorphism, Q192R, determines whether glutamine (Q) or arginine (R) is present near PON1’s catalytic center (Harel et al. 2004). The two resulting PON1_192_ isoforms hydrolyze some substrates at different rates. In mice, PON1_R192_ isoform provides significantly better protection than does PON1_Q192_ from chlorpyrifos oxon (Li et al. 2000). The two isoforms provide similar protection with respect to the oxon of diazinon.

Because chlorpyrifos and diazinon are common in children’s environments (Andrew Clayton et al. 2003) and *PON1* genotype may influence susceptibility to these OPs (Cole et al. 2003), we examined whether the inefficient *PON1* promoter allele (*PON1*_-108T_) or the allele coding for the PON1_192_ isoform that may provide lower chlorpyrifos protection (*PON1*_192Q_) are associated with increased risk of CBT in children.

## Materials and Methods

### Subject identification and specimen collection.

Institutional review board approvals were received from the Fred Hutchinson Cancer Research Center and Washington State Department of Health (WDOH) before the conduct of this study. Most subjects were drawn from a previous case–control study (Gurney et al. 1996), with additional control subjects randomly selected from similar birth years. Briefly, in the previous study, cases were diagnosed with primary tumors of the brain, cranial nerves, or meninges [*International Classification of Diseases for Oncology* (ICD-O; World Health Organization [WHO] 1976), codes 191.0–192.1] at < 20 years of age in 1984–1991 while residing in the Seattle–Puget Sound region of Washington State covered by a population-based cancer incidence registry affiliated with the National Cancer Institute’s Surveillance, Epidemiology, and End Results Program. Controls from the same counties were identified via random digit dialing (RDD), frequency matched to cases 2:1 by sex and age. Mothers of all participating children [134 (74%) eligible cases and 281 (79%) eligible controls] were interviewed using a structured questionnaire that included questions about home pesticide treatment, defined as chemical treatment of the house for pests such as termites, fleas, ants, cockroaches, or silverfish, during the index pregnancy and/or childhood up to the diagnosis date (cases) or similar reference date (controls).

A subset of these subjects [70 (52%) cases and 160 (57%) controls] were eligible for the present study because the child was born after 1977 and the mother resided in Washington when the child was born, so a dried blood spot (DBS) card potentially remained archived at the WDOH Newborn Screening Program. Blind to case status, staff located cards and clipped a DBS for 66 (94%) eligible cases and 137 (86%) eligible controls. Most (74%) of the unlocated subjects were born either in the earliest, uncatalogued months (14 of 27) or outside a civilian hospital (6 of 27). Case–control, race/ethnicity, and histologic tumor type proportions from the previous study were preserved in the subset for whom DBS were available. Subjects’ DBS were permanently anonymized (American Society of Human Genetics 1996) before removal from WDOH. DBS from a pilot study that randomly sampled 100 anonymous infants from the same archives and a similar range of birth years (1980–1991) were available to supplement our control group.

### DNA extraction and genotyping.

DNA extraction and genotyping were conducted at the Center for Ecogenetics and Environmental Health Functional Genomics Laboratory at the University of Washington, blind to case status. Six 3-mm punches were removed from each DBS, with all instrumentation flame sterilized between specimens. DNA was then extracted using the QIAamp DNA Mini Kit (QIAGEN, Valencia, CA) according to the manufacturer’s DBS protocol.

*PON1* C-108T and Q192R variants were identified with TaqMan detection system-based assays (respective probes from Integrated DNA Tehcnologies, Coralville, IA, and Applied Biosystems, Foster City, CA). Genotypes were assigned based on relative fluorescence, verified by sequencing as needed. Negative controls and DNA-sequenced positive controls representing each possible genotype were included in each batch of analyses. We included blind duplicate or quadruplicate specimens for 6% of cases and 6% of interviewed controls. All *PON1* genotypes were represented, and the results were in complete agreement with the original specimens. In addition, the laboratory repeated the assays for > 10% randomly selected specimens, also in full agreement.

*PON1* genotyping was completed for all subjects. One control for whom we collected two equally well-matching DBS was excluded from our CBT–*PON1* analyses because *PON1* genotype was different for the two possible matches. Thus, 66 cases and 236 controls were available for statistical analysis.

### Statistical analysis.

Genotype and allele frequencies were tabulated, and chi-square tests were used to check Hardy-Weinberg equilibrium. Using Intercooled Stata (version 8.0; Stata Corp., College Station, TX), we conducted logistic regression to estimate odds ratios (ORs) and 95% confidence intervals (CIs) for CBT in relation to *PON1* genotype (Breslow and Day 1980). Because *PON1* heterozygotes have an intermediate phenotype (Brophy et al. 2001; Leviev and James 2000), each polymorphism was modeled linearly (0, 1, or 2 *PON1*_-108T_ alleles; 0, 1, or 2 *PON1*_192Q_ alleles). To allow for possible threshold effects (e.g., any vs. no PON1_R192_ isoform), the appropriateness of the linear assumption was verified using the likelihood-ratio test and by comparing modeled risk estimates to those obtained for individual genotypes. Test of trend *p*-values for CBT and number of *PON1*_-108T_ or *PON1*_192Q_ alleles were derived from the linear terms in the logistic regression models.

To consider the possible combined effect of the two polymorphisms, we investigated whether risk estimates for one polymorphism depended on the other and calculated CBT risk estimates in relation to *PON1*_C-108T/Q192R_ diplotypes and haplotypes. For the latter, we modeled the number of each allele (TQ, TR, CQ, CR) linearly, using all subjects for whom haplotype could be directly inferred or accurately estimated (PHASE, version 2.0.2) (Stephens et al. 2001). We also used PHASE to test whether cases and controls had different haplotype frequencies.

CBT (Gurney et al. 1999) and the *PON1*_-108T_ and *PON1*_192Q_ alleles (Brophy et al. 2002; Chen et al. 2003) are more common in non-Hispanic whites than in individuals of most other races or ethnicities. To investigate whether population stratification influenced risk estimates, we repeated all analyses restricted to children whose biologic mother and father were both non-Hispanic white. Race/ethnicity was not otherwise included in models because there were few nonwhite or Hispanic subjects, and within this heterogeneous category for which prevalence of *PON1* variants varies widely, there were substantial racial/ethnic differences between cases and controls.

Other potential confounders considered were the other *PON1* polymorphism (C-108T by Q192R, and vice versa) and the frequency-matching variables (sex and age). These were retained in the models only if ORs or 95% CIs for the *PON1* polymorphisms were altered by > 10%, and unless stated, unadjusted risk estimates are presented. We also examined whether CBT–*PON1* risk estimates varied by reported home pesticide treatment, farm residence, or parental agricultural occupation, potential indicators of exposure to OPs metabolized by PON1. Statistical interaction on the multiplicative scale was assessed in logistic regression models by the *p*-value (α= 0.05) of the interaction term, or likelihood-ratio test when a single interaction required multiple terms. To the extent possible, we calculated risk estimates by histologic subtype: astroglial tumors (ICD-O histology codes 9380–9384, 9400–9421, 9424–9442), primitive neuroectodermal tumors (PNETs; 9362, 9470–9473, 9500), and other CBTs, using all controls as the reference group (WHO 1976).

## Results

### Subject characteristics.

Approximately half of cases had astroglial tumors, with the remainder evenly divided between PNETs and a heterogeneous group of other tumors (Table 1). Most cases (71%) were diagnosed before the age of 5 years. Similar proportions of cases (88%) and controls (91%) were non-Hispanic white; however, among subjects for whom the father’s race/ethnicity was known (63 cases, 135 interviewed controls), proportionally fewer cases (81%) than controls (93%) were born to two non-Hispanic white parents.

Proportionally fewer mothers of cases (31%) than controls (42%) reported that any of the child’s homes had ever been chemically treated for pests during the pregnancy or childhood before the diagnosis/reference date. In this largely urban/suburban region, few subjects had ever lived on a farm (9% cases, 3% controls) or had parents who worked in agriculture (14% cases, 10% controls).

### PON1 *genotype*.

*PON1* genotype frequencies did not significantly differ from Hardy-Weinberg equilibrium (both *p* > 0.20). Proportionally more cases (26%) than controls (17%) were homozygous for the inefficient *PON1* promoter allele (*PON1*_-108T_), and risk of CBT was nonsignificantly increased with increasing *PON1*_-108T_ alleles (for *PON1*_-108TT_, relative to *PON1*_-108CC_: OR = 2.1; 95% CI, 0.9–4.7; for *PON1*_-108CT_, OR = 1.4; 95% CI, 1.0–2.2; *p*-value for trend = 0.07; Table 2). The association was strongest and statistically significant in relation to the PNET histologic tumor type specifically (for each additional *PON1_-_*_108T_ allele: OR = 2.4; 95% CI, 1.1–5.4; *p*-value for trend = 0.03, based on 15 PNET cases, including 6 *PON1*_-108TT_ homozygotes and 7 heterozygotes; data not shown). According to *PON1*_192_ genotype, 48% cases and 42% controls had no PON1_R192_ isoform (Table 2). Although there was a weak suggestion of increased CBT risk in relation to increasing number of *PON1*_192Q_ alleles, CIs were quite wide and the *p*-value for trend was nonsignificant (for *PON1*_192QQ_, relative to *PON1*_192RR_: OR = 1.5; 95% CI, 0.6–3.4; for *PON1*_192QR_: OR = 1.2; 95% CI, 0.8–1.9; *p-*value for trend = 0.36). None of the above risk estimates was attenuated when we restricted the analysis to children with two non-Hispanic white parents, nor were they markedly altered when we separately used either the interviewed controls identified through RDD or the anonymous WDOH archive controls as the reference group.

Logistic regression models indicated no interaction (*p* = 0.75) between these two *PON1* polymorphisms. Indeed, we observed a positive association between CBT and the *PON1*_-108T_ allele within each *PON*_192_ geno-type, and the estimated risk per *PON1*_-108T_ allele was nearly identical in children with (OR = 1.5; 95% CI, 0.9–2.6) or without (OR = 1.4; 95% CI, 0.8–2.5) any PON1_R192_ isoform (data not shown). However, with respect to CBT–*PON1*_Q192R_, the possible weak association was absent among *PON1*_-108TT_ homozygotes (per *PON1*_192Q_ allele relative to *PON1*_192RR_: OR = 1.0; 95% CI, 0.4–2.8.

### PON1 *diplotype and haplotype.*

We observed proportionally more cases than controls in each *PON1*_C-108T/Q192R_ diplotype with two inefficient *PON1* promoters and in the CT/QQ diplotype (no PON1_R192_ isoform and only one efficient promoter allele; data not shown). Proportionally fewer cases were represented in each of the other diplo-types, those with two efficient promoters or those with only one efficient promoter but some PON1_R192_ isoform.

*PON1*_C-108T/Q192R_ haplotype frequencies were not significantly different in cases versus controls (*p* = 0.09). The haplotype model confirmed the earlier impression that the *PON1*_192Q_ allele was not associated with CBT among *PON1-*_108TT_ homozygotes: the risk of CBT relative to children with the CR/CR haplotype (homozygous for efficient promotion of PON1_192R_ isoform) was 1.7 (95% CI, 1.0–3.2) per TQ allele, 1.7 (95% CI, 0.6–4.3) per TR allele, and 1.3 (95% CI, 0.7–2.6) per CQ allele. However, the resulting risk estimates for individual diplotypes were not markedly different from those estimated by simpler models, including one with only a single linear term for each polymorphism.

### PON1 *and pesticide exposure indicators.*

CBT risk was associated with *PON1*_C-108T_ only among children whose mothers reported that at least one of the child’s homes had been chemically treated for pests. Relative to *PON1*_-108CC_, the risk per *PON1*_-108T_ allele was 2.6 (95% CI, 1.2–5.5), whereas among children whose homes were reportedly not treated, the risk was 0.9 (95% CI, 0.5–1.6; interaction *p* = 0.03; Table 3). Any suggestion of an interaction between *PON1*_Q192R_ and home pesticide treatment was not statistically significant (*p* = 0.33; Table 4). These observations did not appear to be caused by case–control differences in demographic factors associated with pesticide use/reporting, such as race/ethnicity, maternal education, or smoking. Our ability to examine the CBT–*PON1* relation by farm residence or parental agricultural occupation was quite limited, although there was a higher frequency of *PON1*_-108TT_ cases among children who had lived on a farm (4 of 6 cases, 0 of 4 controls). Combining all three pesticide exposure indicators, the risk of CBT per *PON1*_-108T_ allele was 2.0 (95% CI, 1.03–3.7) among exposed and 1.0 (95% CI, 0.5–1.9) among unexposed (interaction *p* = 0.15; data not shown.)

## Discussion

This small population-based study suggests that having an inefficient *PON1* promoter allele at position –108 is associated with an increased risk of CBT. The observed association was strongest with respect to PNET, the CBT type most consistently associated with farm residence (Bunin et al. 1994; Kristensen et al. 1996). For the most part, CBT was not associated with the *PON1*_Q192R_ polymorphism, which determines the enzyme’s structure and thereby detoxification efficiency for some substrates. Our results were similar when we focused on the largest racial/ethnic group in our population, indicating that potential biases related to race/ethnicity were probably not largely responsible for our observations. In addition, risk estimates were fairly resilient to exclusion of either of the two population-based sources of controls. Nevertheless, any association between CBT and *PON1*_C-108T_, and the lack thereof between CBT and *PON1*_Q192R_, must be interpreted with caution.

First, our small numbers of subjects may have hampered our ability to observe a CBT–*PON1*_Q192R_ association and simultaneously increased the probability that the apparent association between CBT and *PON1*_C-108T_ is a false positive. Furthermore, no prior studies have examined this relationship. Non-Hodgkin lymphoma (Kerridge et al. 2002), multiple myeloma (Lincz et al. 2004), and prostate cancer (Antognelli et al. 2004; Marchesani et al. 2003) have been associated with other *PON1* polymorphisms, including Q192R, but to our knowledge this is the first cancer study to consider the C-108T polymorphism of *PON1*, and the first study to examine the potential role of this enzyme in relation to childhood cancer.

Second, we obtained subjects’ DNA from an indirect source. It is possible that environmental DNA contamination occurred, which would likely bias risk estimates toward null. However, our assays do not simply detect the presence of an allele, but instead rely on relative amounts of each allele compared with sequence-verified laboratory controls to assign one of three genotypes.

Third, although our specimen retrieval rate for cases was very high and did not require cases to have survived, we cannot rule out survival-related selection bias among cases in the study from which they were drawn. However, even if such bias existed and *PON1* genotype influenced cancer prognosis, it is unlikely this could account fully for the moderately strong association we observed between CBT and *PON1*_C-108T_.

To the extent that this association is not due to chance or case survival, these results suggest that CBT risk may be inversely related to PON1 enzyme levels. Ideally, fresh blood would have been available and PON1 enzyme levels measured, but the C-108T polymorphism is a significant determinant of PON1 levels in both neonates and pregnant women (Chen et al. 2003). Because PON1 may hydrolyze OPs before they can reach the brain, our results lend support to prior epidemiologic studies that have observed an increased risk of CBT in relation to possible pesticide exposure. OPs target the developing nervous system (Garcia et al. 2002; Johnson et al. 1998), and chlorpyrifos may affect replication and differentiation of glial cells (Garcia et al. 2001).

If OPs do relate to CBT risk, one would expect CBT–*PON1* associations to be present mainly among those exposed to chlorpyrifos and/or diazinon. These two OPs were the most common residential insecticides during the study years, and indoor air is an important source of children’s exposure to them (Andrew Clayton et al. 2003). Therefore, it is interesting that we observed an increased risk of CBT in relation to the inefficient *PON1*_-108T_ allele only among children whose homes were reportedly treated for insect pests. Still, one cannot discount the possibility that this OP-metabolizing enzyme could protect the brain via its more generic ability to metabolize oxidized lipid molecules. That *PON1*_Q192R_ was not associated with CBT seems to underscore this point. However, the relative protection provided by the two resulting PON1_192_ isoforms depends on the OP (Li et al. 2000), and perhaps other factors such as PON1 levels. We had no direct measures of these, nor of the level of exposure to chlorpyrifos and diazinon. Because of our modest number of cases, we also had limited ability to consider other potentially relevant factors, such as diet, age at diagnosis, family history of cancer, and prenatal and childhood exposure to tobacco smoke. For example, there was some indication that the *PON1*_192Q_ allele is associated with increased risk of CBT among subjects reportedly not exposed to tobacco smoke, but we could not adequately examine whether this reflects a plausible biologic effect or is simply a spurious observation due to chance or bias related to racial/ethnic differences between cases and controls and smokers and nonsmokers.

The strengths of our study are population-based identification of cases and controls, the use of a DNA source unrelated to case survival, and inclusion of children diagnosed before the residential phase-out of chlorpyrifos and diazinon began. Future studies of CBT and PON1 would benefit from larger sample sizes, more accurate indicators of exposure to chlorpyrifos and diazinon, the addition of other *PON* polymorphisms, measurement of plasma PON1 activity (e.g., PON1 status) (Costa and Furlong 2002), and genotyping/phenotyping of both children and mothers. It would also be useful to know whether the *PON1*_C-108T_ polymorphism is associated with biomarkers of relevance to cancer, such as chromosome aberrations, as has been demonstrated in farmers with respect to *PON1*_Q192R_ (Au et al. 1999). Such studies would be worthwhile in light of the possible association we observed between CBT occurrence and the predominant polymorphism in the *PON1* promoter region, and because chlorpyrifos remains a leading agricultural insecticide and is detected in or on foods frequently consumed by children (Andrew Clayton et al. 2003; U.S. Department of Agriculture 2004).

## Figures and Tables

**Table 1:** Demographic characteristics and selected exposures among children with and without brain tumors [*n* (%)].

	Cases (*n* = 66)	Interviewed controls (*n* = 136)	Anonymous controls (*n* = 100)	All controls (*n* = 236)
Race/ethnicity*^a^*				
White	58 (88)	131 (96)	33 (75)	164 (91)
Black	0 (0)	2 (1)	4 (9)	6 (3)
Hispanic	0 (0)	1 (1)	5 (11)	6 (3)
Asian	3 (5)	0 (0)	2 (5)	2 (1)
Other	5 (8)	2 (1)	0 (0)	2 (1)
Male	42 (64)	84 (62)	57 (58)*^b^*	141 (60)*^b^*
Birth year				
1978–1983	30 (45)	71 (52)	50 (50)	121 (51)
1984–1991	36 (55)	65 (48)	50 (50)	115 (49)
Age at diagnosis/reference (years)				
< 5	47 (71)	89 (65)	—	—
5–10	19 (29)	47 (35)	—	—
Mother smoked during pregnancy	10 (15)	23 (17)	—	—
Pesticide exposure indicators				
Home pesticide treatment*^c^*	20 (31)*^d^*	57 (42)	—	—
Farm residence*^c^*	6 (9)	4 (3)	—	—
Parental agricultural occupation*^e^*	9 (14)	14 (10)	—	—
Histologic tumor type				
Astroglial	37 (56)	—	—	—
PNET	15 (23)	—	—	—
Other	14 (21)	—	—	—

—, data not available.

a As determined by maternal interview (cases and interviewed controls) or checkbox for child on DBS card (anonymous controls; not available for births before 1990); percentage excludes 56 anonymous controls for whom race/ethnicity was not reported.

b Percentage excludes two anonymous controls for whom sex was not reported on the DBS card.

c During pregnancy or childhood before diagnosis/reference date.

d Percentage excludes one case for whom pesticide use was unknown.

e In year of birth or prior 4 years: agricultural occupation/industry (Cordier et al. 2001), or occupational exposure to pesticides/weedkillers, fertilizer, “other” agricultural chemicals, farm animals, manure, or other potential indicators of chlorpyrifos/diazinon contact (domestic animals/birds for resale, unprocessed wool, hides/skins/feathers, or “other” animal products, excluding raw meat and milk).

**Table 2:** Risk of CBT in relation to *PON1* C-108T and Q192R polymorphisms [*n* (%)].

	All subjects			Subjects with white parents*^a^*		
*PON1* genotype	Cases (*n* = 66)	Controls (*n* = 236)	OR (95% CI)*^b^*	Cases (*n* = 51)	Controls (*n* = 125)	OR (95% CI)*^b^*
C-108T: PON1 promotion						
TT (inefficient)	17 (26)	39 (17)	2.1 (0.9–4.7)	14 (27)	22 (18)	2.6 (1.0–6.9)
CT (intermediate)	34 (52)	125 (53)	1.4 (1.0–2.2)	28 (55)	66 (53)	1.6 (1.0–2.6)
CC (efficient)	15 (23)	72 (31)	1.0 (reference)	9 (18)	37 (30)	1.0 (reference)
Q192R: PON1_R192_ isoform						
QQ (none)	32 (48)	100 (42)	1.5 (0.6–3.4)*^c^*	27 (53)	58 (46)	1.6 (0.5–4.6)
QR (some)	28 (42)	105 (44)	1.2 (0.8–1.9)*^c^*	21 (41)	57 (46)	1.3 (0.7–2.1)
RR (all)	6 (9)	31 (13)	1.0 (reference)	3 (6)	10 (8)	1.0 (reference)

a Biologic mother and father both reportedly non-Hispanic and white; excludes 3 cases, 1 interviewed control, and 100 anonymous controls for whom father’s race/ethnicity unknown.

b For individual genotypes, with each polymorphism modeled linearly (0, 1, or 2 *PON1*_-108T_ alleles; 0, 1, or 2 *PON1*_192Q_ alleles) using logistic regression.

c Adjusted for *PON1*_C-108T_.

**Table 3:** Risk of CBT in relation to *PON1*_C-108T_ genotype, by ever/never chemical treatment of home for insect pests during pregnancy or childhood.

Home pesticide treatment*^a^*	*PON1*_C-108T_ genotype	Cases [*n* = 65*^b^* (%*^c^*)]	Controls [*n* = 136*^d^* (%*^c^*)]	OR (95% CI)*^e^*	OR (95% CI)*^f^*
Yes	TT	9 (45)	10 (18)	1.4 (0.5–3.9)	6.6 (1.5–29.7)
	CT	8 (40)	26 (46)	0.5 (0.2–1.3)	2.6 (1.2–5.5)
	CC	3 (15)	21 (37)	0.2 (0.1–0.7)	1.0 (reference)
No	TT	7 (16)	13 (16)	0.8 (0.3–2.7)	
	CT	26 (58)	47 (59)	0.9 (0.5–1.6)	
	CC	12 (27)	19 (24)	1.0 (reference)	

a Based on maternal interview; chemical treatment of home for termites, fleas, cockroaches, ants, silverfish, or other, during index pregnancy or during childhood before diagnosis/reference date.

b Excludes one case for whom home pesticide treatment was unknown.

c Within respective pesticide category.

d Excludes anonymous controls, due to absence of pesticide interview data.

e Relative to *PON1*_-108CC_ homozygotes whose homes were not chemically treated for insect pests.

f Relative to *PON1*_-108CC_ homozygotes whose homes were chemically treated for insect pests.

**Table 4:** Risk of CBT in relation to *PON1*_Q192R_ genotype, by ever/never chemical treatment of home for insect pests during pregnancy or childhood.

Home pesticide treatment*^a^*	*PON1*_Q192R_ genotype	Cases [*n* = 65*^b^* (%*^c^*)]	Controls [*n* = 136*^d^* (%*^c^*)]	OR (95% CI)*^e^*	OR (95% CI)*^f^*
Yes	QQ	8 (40)	26 (46)	0.8 (0.2–2.4)	0.6 (0.1–3.3)
	QR	10 (50)	27 (47)	1.0 (0.3–2.7)	0.8 (0.4–1.8)
	RR	2 (10)	4 (7)	1.2 (0.3–5.2)	1.0 (reference)
No	QQ	23 (51)	33 (42)	1.7 (0.6–5.4)	
	QR	18 (40)	37 (47)	1.3 (0.7–2.3)	
	RR	4 (9)	9 (11)	1.0 (reference)	

a Based on maternal interview; chemical treatment of home for termites, fleas, cockroaches, ants, silverfish, or other, during index pregnancy or during childhood before diagnosis/reference date.

b Excludes one case for whom home pesticide treatment was unknown.

c Within respective pesticide category.

d Excludes anonymous controls, due to absence of pesticide interview data.

e Relative to *PON1*_192RR_ homozygotes whose homes were not chemically treated for insect pests.

f Relative to *PON1*_192RR_ homozygotes whose homes were chemically treated for insect pests.
